# Mini-PEG spacering of VAP-1-targeting ^68^Ga-DOTAVAP-P1 peptide improves PET imaging of inflammation

**DOI:** 10.1186/2191-219X-1-10

**Published:** 2011-07-26

**Authors:** Anu Autio, Tiina Henttinen, Henri J Sipilä, Sirpa Jalkanen, Anne Roivainen

**Affiliations:** 1 Turku PET Centre, University of Turku and Turku University Hospital, Turku, Finland; 2 Department of Biology, Division of Genetics and Physiology, University of Turku, Turku, Finland; 3 MediCity Research Laboratory, University of Turku, Turku, Finland; 4 Turku Center for Disease Modeling, University of Turku, Turku, Finland

**Keywords:** gallium-68, inflammation imaging, mini-PEG spacer, positron emission tomography, vascular adhesion protein-1

## Abstract

**Background:**

Vascular adhesion protein-1 (VAP-1) is an adhesion molecule that plays a key role in recruiting leucocytes into sites of inflammation. We have previously shown that ^68^Gallium-labelled VAP-1-targeting peptide (^68^Ga-DOTAVAP-P1) is a positron emission tomography (PET) imaging agent, capable of visualising inflammation in rats, but disadvantaged by its short metabolic half-life and rapid clearance. We hypothesised that prolonging the metabolic half-life of ^68^Ga-DOTAVAP-P1 could further improve its imaging characteristics. In this study, we evaluated a new analogue of ^68^Ga-DOTAVAP-P1 modified with a mini-polyethylene glycol (PEG) spacer (^68^Ga-DOTAVAP-PEG-P1) for *in vivo *imaging of inflammation.

**Methods:**

Whole-body distribution kinetics and visualisation of inflammation in a rat model by the peptides ^68^Ga-DOTAVAP-P1 and ^68^Ga-DOTAVAP-PEG-P1 were evaluated *in vivo *by dynamic PET imaging and *ex vivo *by measuring the radioactivity of excised tissues. In addition, plasma samples were analysed by radio-HPLC for the *in vivo *stability of the peptides.

**Results:**

The peptide with the mini-PEG spacer showed slower renal excretion but similar liver uptake as the original peptide. At 60 min after injection, the standardised uptake value of the inflammation site was 0.33 ± 0.07 for ^68^Ga-DOTAVAP-P1 and 0.53 ± 0.01 for ^68^Ga-DOTAVAP-PEG-P1 by PET. In addition, inflammation-to-muscle ratios were 6.7 ± 1.3 and 7.3 ± 2.1 for ^68^Ga-DOTAVAP-P1 and ^68^Ga-DOTAVAP-PEG-P1, respectively. The proportion of unchanged peptide in circulation at 60 min after injection was significantly higher for ^68^Ga-DOTAVAP-PEG-P1 (76%) than for ^68^Ga-DOTAVAP-P1 (19%).

**Conclusion:**

The eight-carbon mini-PEG spacer prolonged the metabolic half-life of the ^68^Ga-DOTAVAP-P1 peptide, leading to higher target-to-background ratios and improved *in vivo *PET imaging of inflammation.

## Background

*In vivo *imaging of inflammation is a demanding task, and novel molecular imaging targets are called for. The gold standard in nuclear medicine is the radiolabelling of white blood cells, which is both time consuming and potentially hazardous for the technical personnel.

Vascular adhesion protein-1 (VAP-1) is an inflammation-inducible endothelial adhesion protein involved in the leucocyte trafficking from the blood stream into the tissues. VAP-1 is stored in intracellular granules within endothelial cells. However, upon inflammation, it is rapidly translocated to the endothelial cell surface, for example, in the synovial tissue in rheumatoid arthritis and at the site of ischemic reperfusion injury [1,2]. Therefore, VAP-1 is both an optimal candidate for anti-inflammatory therapy and a potential target for *in vivo *imaging of inflammation. This approach may open new opportunities for diagnosing, therapy planning and monitoring of the treatment efficacy, as well as for the drug discovery and development processes [3-6].

Peptide-based imaging agents are small molecules that possess favourable properties such as rapid diffusion in target tissue, rapid clearance from the blood circulation and non-target tissues, easy and low-cost synthesis, and low toxicity and immunogenicity. We are particularly interested in developing radiolabelled peptides for VAP-1 targeting for the purposes of *in vivo *imaging of leucocyte trafficking. The linear peptide, VAP-P1, has been characterised by Yegutkin et al. and proven to bind the enzymatic groove of VAP-1 and dose-dependently inhibit VAP-1-dependent lymphocyte rolling and firm adhesion to primary endothelial cells [7]. We have previously shown that ^68^Ga-labelled DOTA-conjugated VAP-P1 peptide (^68^Ga-DOTAVAP-P1) is able to delineate inflammation in rats by a VAP-1-specific way using positron emission tomography (PET) [8-10]. Disadvantageously, the ^68^Ga-DOTAVAP-P1 peptide has relatively short plasma half-life and very rapid clearance by the kidneys to the urine.

PEGylation, the process by which polyethylene glycol (PEG) chains or its derivatives, e.g., mini-PEGs are attached to a peptide, has been used for modifying the properties of radiolabelled compounds, such as antibodies and peptides, in order to improve their imaging characteristics. The goal of PEGylation is mainly to improve the tracer's kinetics and distribution pattern by increasing its metabolic half-life and by lowering its non-specific binding. By increasing the molecular mass of the peptide and by shielding it from proteolytic enzymes, PEGylation may modify its biodistribution and pharmacokinetics [11]. Thus, the method could overcome the above mentioned shortcomings. However, because PEGylation may also have unfavourable effects, such as inhibition of receptor binding and reduction of target-to-background ratio, its impact must be carefully evaluated for a new peptide.

We hypothesised that prolonging the metabolic half-life of ^68^Ga-DOTAVAP-P1 would further improve its potential for *in vivo *imaging of inflammation. In this study, we evaluated a new mini-PEG spacered analogue of ^68^Ga-DOTAVAP-P1 (^68^Ga-DOTAVAP-PEG-P1) for *in vivo *PET imaging of inflammation.

## Methods

### ^68^Ga-DOTA-peptides

The DOTA-conjugated peptides were purchased from Almac Sciences (By Gladsmuir, Scotland, UK), ABX advanced biochemical compounds GmbH (Radeberg, Germany) and NeoMPS (Strasbourg, France).

Linear 9-amino acid DOTA-chelated peptide (GGGGKGGGG) with and without a PEG linker (8-amino-3,6-diooxaoctanoyl, PEG derivative, MW 145.16 Da) between the DOTA and the N terminal amino acid was labelled with ^68^Ga as previously described [8], and named as ^68^Ga-DOTAVAP-P1 and ^68^Ga-DOTAVAP-PEG-P1. Briefly, ^68^Ga was obtained in the form of ^68^GaCl_3 _from a ^68^Ge/^68^Ga generator (Cyclotron Co., Obninsk, Russia) by elution with 0.1 M HCl. The ^68^GaCl_3 _eluate (500 μl) was mixed with sodium acetate (18 mg; Sigma-Aldrich, Seelze, Germany) to give a pH of approximately 5.5. Then, DOTA-peptide (35 nmol) was added and the mixture was incubated at 100°C for 20 min. No further purification was needed.

The radiochemical purity was determined by reversed-phase HPLC (μBondapak C18, 7.8 × 300 mm^2^, 125 Å, 10 μm; Waters Corporation, Milford, MA, USA). The HPLC conditions for ^68^Ga-DOTAVAP-P1 have been described previously [9]. The HPLC conditions for ^68^Ga-DOTAVAP-PEG-P1 were slightly different and as follows: flow rate = 4 ml/min, *λ *= 215 nm, *A *= 2.5 mM trifluoroacetic acid, *B *= acetonitrile and C = 50 mM phosphoric acid. Linear *A*/*B*/*C *gradient was 100/0/0 for 0 to 3 min, 40/60/0 for 3 to 9 min, and 0/0/100 for 9 to 16 min. The radio-HPLC system consisted of LaChrom instruments (Hitachi; Merck, Darmstadt, Germany): pump L7100, UV detector L-7400 and interface D-7000; an on-line radioisotope detector (Radiomatic 150 TR, Packard, Meriden, CT, USA); and a computerised data acquisition system.

### *In vitro *stability and solubility

The *in vitro *stability of the ^68^Ga-labelled peptides was evaluated in human and rat plasma. Several samples were taken during the 4-h incubation period at 37°C. Proteins from plasma samples were precipitated with 10% sulphosalicylic acid (1:1 *v*/*v*), centrifuged at 3,900 × *g *for 3 min at 4°C, and filtered through 0.45-μm Minispike filter (Waters Corporation). The filtrate was analysed by radio-HPLC.

The octanol-water distribution coefficient, log*D*, of the ^68^Ga-DOTA-peptides was determined using the following procedure. Approximately 5 kBq of ^68^Ga-labelled peptide in 500 μl of phosphate-buffered saline (PBS, pH 7.4) was added to 500 μl of 1-octanol. After the mixture had been vortexed for 3 min, it was centrifuged at 12,000 × *g *for 6 min, and 100-μl aliquots of both layers were counted in a gamma counter (1480 Wizard 3″ Gamma Counter; EG&G Wallac, Turku, Finland). The test was repeated three times. The log*D *was calculated as = log10 (counts in octanol/counts in PBS).

### Animals

All animal experiments were approved by the Lab-Animal Care & Use Committee of the State Provincial Office of Southern Finland and carried out in compliance with the Finnish laws relating to the conduct of animal experimentation.

Male Sprague-Dawley rats (*n *= 14) were purchased from Harlan, Horst, The Netherlands. Twenty-four hours before the PET studies, turpentine oil (Sigma-Aldrich; 0.05 ml per rat) was injected subcutaneously into their neck area in order to induce a sterile inflammation [10]. Six rats were PET imaged and additional eight animals were used for *in vivo *metabolite analyses.

### PET imaging and *ex vivo *biodistribution

The whole-body distribution and kinetics of ^68^Ga-DOTAVAP-P1 (*n *= 3) and ^68^Ga-DOTAVAP-PEG-P1 (*n *= 3) in rats harbouring a sterile inflammation were studied with a high-resolution research tomograph (Siemens Medical Solutions, Knoxville, TN, USA). The rats were anaesthetised with isoflurane (induction 3%, maintenance 2.2%). Two rats were imaged at the same time, and they were kept on a warm pallet during the imaging procedure. Following a 6-min transmission for attenuation correction, the rats were intravenously (i.v.) injected with ^68^Ga-DOTAVAP-P1 (15.8 ± 3.0 MBq, 19.4 ± 0.0 μg, 19.6 ± 0.0 nmol) or with ^68^Ga-DOTAVAP-PEG-P1 (17.7 ± 1.6 MBq, 21.0 ± 1.3 μg, 18.5 ± 1.1 nmol) as a bolus via a tail vein using a 24-gauge cannula (BD Neoflon, Becton Dickinson Infusion Therapy AB, Helsingborg, Sweden). Dynamic imaging lasting for 60 min started at the time of injection. The data acquired in list mode were iteratively reconstructed with a 3-D ordered subsets expectation maximisation algorithm with 8 iterations, 16 subsets and a 2-mm full-width at half-maximum post-filter into 5 × 60 s and 11 × 300 s frames. Quantitative analyses were performed by drawing regions of interest (ROI) on the inflammatory foci, muscle (hind leg), heart, kidney, liver and urinary bladder. Time-activity curves (TACs) were extracted from the corresponding dynamic images (Vinci software, version 2.37; Max Planck Institute for Neurological Research, Cologne, Germany). The average radioactivity concentrations in the ROIs (kilobecquerels per millilitre) were used for further analyses. The uptake was reported as standardised uptake value (SUV), which was calculated as the radioactivity of the ROI divided by the relative injected radioactivity expressed per animal body weight. The radioactivity remaining in the tail was compensated.

After the PET imaging, the animals were sacrificed. Samples of blood, urine and various organs were collected, weighed and measured for radioactivity using the gamma counter (Wizard, EG&G Wallac). The results were expressed as SUVs.

### Blood analyses

Blood samples (0.2 ml of each) were drawn at 5, 10, 15, 30, 45, 60 and 120 min after injection of ^68^Ga-DOTA-peptides into heparinised tubes (Microvette 100; Sarstedt, Nümbrecht, Germany). Radioactivity of whole blood was measured with the gamma counter (Wizard, EG&G Wallac). Plasma was separated by centrifugation (2,200 × *g *for 5 min at 4°C), and plasma radioactivity was measured. The ratio of radioactivity in blood versus plasma was calculated. To determine plasma protein binding, proteins were precipitated with 10% sulphosalicylic acid, and the radioactivity in protein precipitate and supernatant was measured. The plasma supernatant was further analysed by radio-HPLC in order to evaluate the *in vivo *stability of the ^68^Ga-labelled peptides.

*In vivo *stability data were used in order to generate metabolite-corrected plasma TACs for ^68^Ga-DOTAVAP-P1 and ^68^Ga-DOTAVAP-PEG-P1, which were further used for the calculation of pharmacokinetic parameters. The area under curve (AUC) of the plasma TAC from 0 to infinity was calculated using a non-compartmental analysis employing the trapezoidal rule. The clearance (CL) of the ^68^Ga-labelled peptides after a single intravenous bolus dose was calculated by dividing the injected dose by the AUC. The plot of the natural logarithm of parent tracer concentration against time after bolus injection became linear in the end phase, as the tracer was eliminated according to the laws of first-order reaction kinetics. The elimination rate constant (*k*_el_) was calculated as the negative slope of the linear part of the plot. The plasma elimination half-life (*t*_1/2_) was calculated as *t*_1/2 _= ln(2)/*k*_el_. The metabolic half-lives of the ^68^Ga-DOTA-peptides were calculated according to the results of radio-HPLC, i.e. the time point when 50% of the total radioactivity is still bound to the intact peptide.

### Statistical analyses

All the results are expressed as means ± standard deviation (SD) and range. The correlations between PET imaging and *ex vivo *measurement values were evaluated using linear regression analysis. Inter-group comparisons were made using an unpaired *t *test. Statistical analyses were conducted using Origin 7.5 software (Microcal, Northampton, MA, USA). A *P *value less than 0.05 was considered as statistically significant.

## Results

### *In vitro *studies

The radiochemical purities of ^68^Ga-DOTAVAP-P1 and ^68^Ga-DOTAVAP-PEG-P1 were 97 ± 1% and 99 ± 1%, and specific radioactivities 2.27 ± 0.47 and 2.55 ± 0.45 MBq/nmol, respectively. The retention times for ^68^Ga-DOTAVAP-P1 and ^68^Ga-DOTAVAP-PEG-P1 were 6.6 ± 0.1 and 6.7 ± 0.1 min, respectively. The retention time for free gallium was approximately 12 min, and it eluted only with phosphoric acid. The *in vitro *stabilities of ^68^Ga-DOTAVAP-P1 and ^68^Ga-DOTAVAP-PEG-P1 were very similar. The amounts of unchanged peptide after the 4-h incubation in human or rat plasma were 88 ± 3% and 82 ± 11% for ^68^Ga-DOTAVAP-P1 and 89 ± 8% and 90 ± 6% for ^68^Ga-DOTAVAP-PEG-P1, respectively. Both peptides were highly hydrophilic; log*D *was -3.30 for ^68^Ga-DOTAVAP-P1 and -3.50 for ^68^Ga-DOTAVAP-PEG-P1.

### PET studies with rat model of inflammation

**Figure 1:**
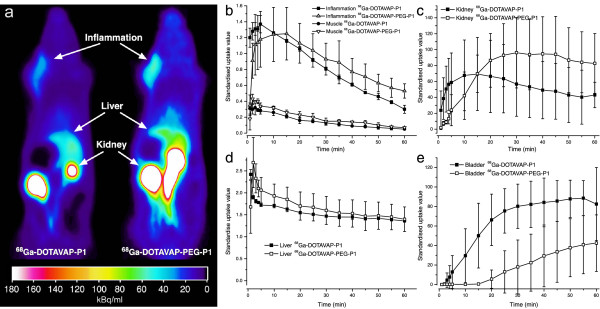
**PET images and time-activity curves**. (**a**) Representative coronal PET images of Sprague-Dawley rats with sterile turpentine oil-induced inflammation as a sum image of 10 to 60 min after i.v. injection of ^68^Ga-DOTAVAP-P1 (13.8 MBq) or ^68^Ga-DOTAVAP-PEG-P1 (17.5 MBq). Time-activity curves of (**b**) inflammation and muscle, (**c**) kidney, (**d**) liver and (**e**) urinary bladder for ^68^Ga-DOTAVAP-P1 and ^68^Ga-DOTAVAP-PEG-P1.

Both peptides were capable of visualising inflammatory foci from surrounding tissues by PET imaging (Figure 1a). The inflammation uptakes expressed as SUVs were 0.33 ± 0.07 (range, 0.26 to 0.40) and 0.53 ± 0.01 (range, 0.42 to 0.60) for ^68^Ga-DOTAVAP-P1 and ^68^Ga-DOTAVAP-PEG-P1, respectively, at 60 min after injection. Inflammation-to-muscle ratios at 60 min after injection were 6.7 ± 1.3 (range, 5.2 to 7.5) and 7.3 ± 2.1 (range, 5.6 to 9.7) for ^68^Ga-DOTAVAP-P1 and ^68^Ga-DOTAVAP-PEG-P1, respectively. The kinetics of ^68^Ga-DOTAVAP-P1 and ^68^Ga-DOTAVAP-PEG-P1 in inflammatory foci were quite fast, and the peak radioactivity was reached within 20 min for both peptides. On the average, the inflammation uptake of ^68^Ga-DOTAVAP-PEG-P1 was 59% higher than that of ^68^Ga-DOTAVAP-P1, and the difference was statistically significant (*P *= 0.047). According to the whole-body dynamic PET imaging, ^68^Ga-DOTAVAP-PEG-P1 showed slower renal excretion to urine but otherwise rather similar distribution kinetics as the original peptide ^68^Ga-DOTAVAP-P1 (Figure 1b, c, d, e).

**Figure 2:**
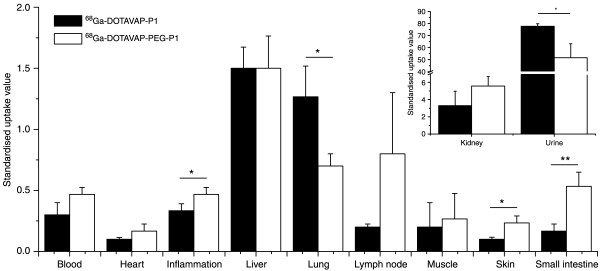
**Biodistribution of the ^68^Ga-DOTA-peptides at 60 min after injection**. Results are given as the mean ± SD of three experiments. Asterisks indicate statistically significant differences between the peptides. *P < 0.05; **P < 0.01.

The PET imaging results were verified by *ex vivo *measurements (Figure 2). Linear regression analysis showed reasonable correlation between *in vivo *PET and *ex vivo *tissue samples (*R *= 0.58, *P *= 0.023 for ^68^Ga-DOTAVAP-P1 and *R *= 0.80, *P *< 0.001 for ^68^Ga-DOTAVAP-PEG-P1). When the tissue uptakes of ^68^Ga-DOTAVAP-P1 and ^68^Ga-DOTAVAP-PEG-P1 were compared, the inflammation, lung, small intestine, skin and urinary bladder radioactivities were significantly different. Although the PET and *ex vivo *methods correlate well, there are some discrepancies between the results. For example, in the PET image analysis, the urine and blood of kidney are included in the "kidney" ROI, whereas for *ex vivo *measurement, the excised tissue samples are dotted dry on a paper. Since the radioactivity of urine is extremely high, the *in vivo *kidney SUV is higher than that of *ex vivo*.

**Figure 3:**
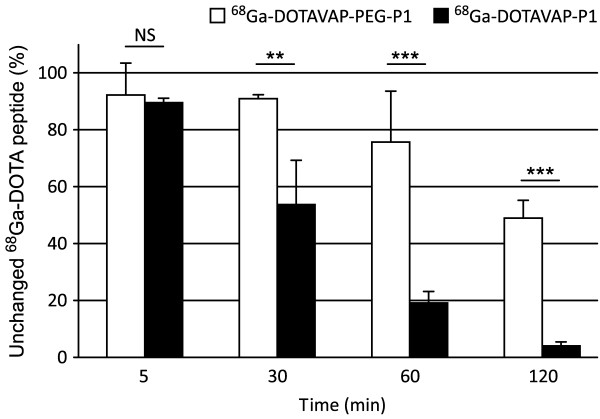
***In vivo *stability of the i.v. administered ^68^Ga-DOTA-peptides in blood circulation of the rat**. Results are given as the means of three to seven experiments. NS, not significant; **P < 0.01; ***P < 0.001.

The blood-plasma ratios and the plasma free fractions (fp), i.e. the fraction of total radioactivity in plasma that is unbound to plasma proteins, were 1.3 ± 0.1 and 0.84 ± 0.04 for ^68^Ga-DOTAVAP-P1 and 1.3 ± 0.1 and 0.86 ± 0.02 for ^68^Ga-DOTAVAP-PEG-P1, respectively. The *in vivo *stability of ^68^Ga-DOTAVAP-PEG-P1 was better than that of ^68^Ga-DOTAVAP-P1. The proportions of unchanged peptides in rat plasma at 60 and 120 min after injection were 19 ± 4% and 4 ± 1% for ^68^Ga-DOTAVAP-P1 and 76 ± 18% and 49 ± 6% for ^68^Ga-DOTAVAP-PEG-P1, respectively (Figure 3). The metabolic half-lives of ^68^Ga-DOTAVAP-P1 and ^68^Ga-DOTAVAP-PEG-P1 were 24 and 125 min, respectively. Based on *in vivo *plasma measurements, ^68^Ga-DOTAVAP-PEG-P1 showed significantly slower *k*_el _and total CL and larger AUC values. In addition, ^68^Ga-DOTAVAP-PEG-P1 had a longer elimination *t*_1/2 _than the original ^68^Ga-DOTAVAP-P1, although the difference was not statistically significant (Table 1).

**Table 1:** Pharmacokinetic parameters of the VAP-1-targeting ^68^Ga-DOTA-peptides

	^68^Ga-DOTAVAP-P1	^68^Ga-DOTAVAP-PEG-P1	*P *value
Elimination *t*_1/2 _(min)	13.4 ± 1.8	28.0 ± 10.7	NS (0.079)
*k*_el _(1/min)	0.05 ± 0.01	0.02 ± 0.01	0.018
AUC (min*kBq/ml)	950 ± 170	2,400 ± 68	0.022
CL (ml/min)	0.017 ± 0.001	0.008 ± 0.002	0.006

*k*_el_, elimination rate constant; AUC, area under curve; CL, clearance; NS, not significant.

## Discussion

Previously, we have reported the feasibility of the VAP-1-targeting peptide, ^68^Ga-DOTAVAP-P1, for PET imaging of inflammation in different rat models [8-10]. However, as a limitation, ^68^Ga-DOTAVAP-P1 is cleared very rapidly from circulation and its *in vivo *stability against degradation by enzymes is only moderate. In this study, we showed that the incorporation of a mini-PEG spacer in ^68^Ga-DOTAVAP-P1 enhanced its *in vivo *stability and improved the PET imaging of inflammation.

The animal model used in our experiments involves turpentine oil injection-induced subcutaneous inflammation as described previously [10]. In that study, we were able to show that the H & E staining of the inflamed site demonstrated infiltration of leucocytes and macrophages at the site of inflammation. The abscess centre with few cells, including residual injected oil, exudates and cell debris, was surrounded by an abscess wall. The dermis also appeared to be inflamed. In the present study, inflammation was evaluated in every animal by visually observing the pale colour of inflamed subcutaneous tissue. We performed *in vitro*, *ex vivo *and *in vivo *experiments to evaluate the VAP-1 targeting, inflammation imaging efficacy and pharmacokinetics of ^68^Ga-DOTAVAP-PEG-P1 in comparison to the original ^68^Ga-DOTAVAP-P1. The incorporation of a mini-PEG spacer had no apparent effect on the *in vitro *properties of the VAP-1 binding peptide; both peptides were stable in plasma incubations and their solubility was very similar. However, when i.v. administered, ^68^Ga-DOTAVAP-PEG-P1 showed significantly longer metabolic and elimination half-lives and slower total clearance compared to ^68^Ga-DOTAVAP-P1. Furthermore, our results revealed that while both peptides were able to visualise experimental inflammation by PET imaging, ^68^Ga-DOTAVAP-PEG-P1 showed a higher inflammation-to-muscle ratio than the original ^68^Ga-DOTAVAP-P1. As regards ^68^Ga-DOTAVAP-P1, the results of this study are in line with our previous publications [8-10]. The renal excretion of ^68^Ga-DOTAVAP-PEG-P1 was slower, resulting in a significantly lower urinary bladder radioactivity in comparison to ^68^Ga-DOTAVAP-P1. The liver uptake was rather high for both peptides, which is, at least in part, due to the high number of VAP-1 receptors in the sinusoidal endothelial cells in the liver [12]. Some degradation products of ^68^Ga-DOTA-peptides, such as free ^68^Ga, also tend to accumulate in the liver [13]. Although modification with a mini-PEG spacer generally decreases liver uptake, the two peptides behaved quite similarly in our study, suggesting a VAP-1-specific binding in this tissue.

PEGylation has widely been used for improving the *in vivo *kinetics of pharmaceuticals. However, the results of such modifications depend much on the nature of the lead compound and the choice of PEG linker [14-20]. In most cases, PEGylation of radiopeptides has advantageous effects, such as increased metabolic half-life, decreased kidney uptake, and improved targeting and subsequent improved targeting for high-quality imaging. However, disadvantageous results have also been reported, e.g. the insertion of a long PEG chain may induce a higher liver uptake and reduce receptor binding [16].

In this study, we incorporated an eight-carbon mini-PEG spacer between the DOTA and the VAP-P1 peptide in order to prolong its biological activity. The 8-amino-3,6-dioxaoctanoic acid contains the shortest ether structure possible of PEG with two ethylene oxide units. A similar spacer has previously been used in imaging agents by Burtea et al. [21], Ke et al. [22] and Silvola et al. [23].

Modification with a mini-PEG spacer increased metabolic stability of VAP-1-targeting DOTA-peptide. In addition, it also improved *in vivo *imaging of inflammation suggesting that PEGylation had other highly pronounced *in vivo *effects beyond modification of pharmacokinetics. Although the modification with a mini-PEG spacer increased the target-to-background ratio, the SUV values in the inflamed area were still very low. Thus, further improvement of the tracer is warranted.

## Conclusion

The incorporation of a mini-PEG spacer enhanced the *in vivo *stability and pharmacokinetics of the VAP-1-targeting peptide, thus leading to higher target-to-background ratios and improved *in vivo *PET imaging of experimental inflammation. ^68^Ga-DOTAVAP-PEG-P1 warrants further investigations for its feasibility in PET imaging of inflammation.

## Abbreviations

HPLC, high performance liquid chromatography; H & E, haematoxylin and eosin; MW, molecular weight; PEG, polyethylene glycol; PET, positron emission tomography; VAP-1, vascular adhesion protein-1.

## Competing interests

The authors declare that they have no competing interests.

## Authors' contributions

AA participated in the design of the study, carried out the *in vitro *and *in vivo *PET studies and drafted the manuscript. TH participated in the design of the study and drafted the manuscript. HJS performed the labelling chemistry and participated in *in vitro *studies. AR and SJ conceived the study, participated in its design and coordination and critically revised the manuscript. All authors read and approved the final manuscript.
